# Circumspinal decompression and fusion through a posterior midline incision to treat central calcified thoracolumbar disc herniation: a minimal 2-year follow-up study with reconstruction CT

**DOI:** 10.1007/s00586-013-3054-4

**Published:** 2013-10-05

**Authors:** Ning Liu, Zhongqiang Chen, Qiang Qi, Weishi Li, Zhaoqing Guo

**Affiliations:** Department of Orthopaedics, Peking University Third Hospital, No. 49 North Garden Road, Beijing, 100191 China

**Keywords:** Thoracolumbar disc herniation, Circumspinal decompression

## Abstract

**Purpose:**

There have been several surgical approaches used in the treatment of thoracolumbar disc herniation (TLDH) from T10/11 to L1/2. However, central calcified TLDH cases are still challenging to spine surgeons. The anterior transthoracic approaches and lateral/posterolateral approaches are all essentially performed from one side; thus, the compressive lesion and the dura matter on the other side of the spinal canal are not clearly visualized, predisposing the procedure to incomplete decompression or inadvertent cord manipulation. Moreover, a number of these approaches are technically demanding and require entry into the chest. The purpose of this study was to introduce a new surgical procedure—circumspinal decompression and fusion through a posterior midline incision—for the treatment of central calcified TLDH and to evaluate its surgical outcome.

**Methods:**

In this study, 22 patients (15 males and 7 females; mean age 49 years) with central calcified TLDH underwent this procedure between April 2008 and April 2011. Altogether, 26 discs were excised, with two discs at T10/11, eight discs at T11/12, nine discs at T12/L1 and seven discs at L1/2. Of these patients, 16 returned for final follow-up, with a mean follow-up period of 41 months (range 24–57 months). Clinical outcomes, including operative time, blood loss, perioperative complications, post-operative time of hospitalization, neurological status improvement, extent of decompression, back pain, local spinal curvature and fusion, were investigated. The patients’ neurological status was evaluated by a modified Japanese Orthopedic Association scoring system of 11 points. Fusion and the extent of decompression were evaluated by reconstruction CT at final follow-up.

**Results:**

The mean operative time was 185 min, the mean blood loss was 896 ml and the mean post-operative hospitalization time was 8 days. Four patients suffered perioperative complications, but only two were related to dura violation and none involved the respiratory system. All of the 16 patients who returned for the final follow-up showed improvement, and evidence of improvement was found in five of the other six patients who did not return for final follow-up through telephone interview or earlier follow-up evaluations. Complete decompression was achieved in 12 of the 16 patients who returned for final follow-up. In the 16 patients who returned for final follow-up, back pain was significantly reduced and local spinal curvature remained unaltered. In addition, based on reconstruction CT images, solid fusion was observed in 15 of the 16 patients who returned for final follow-up.

**Conclusions:**

The circumspinal decompression and fusion through a posterior midline incision procedure can be used to treat central calcified TLDH patients with neurological deficits. This method’s greatest advantage is that it is a highly effective and safe procedure for decompression. Although it is a major and destructive procedure, spinal stability was well maintained in most of the cases. In this era when minimally invasive spine surgeries like thoracoscopy have been in an upward trajectory, spine surgeons still should be made aware of this procedure.

## Introduction

Disc herniation occurring at the thracolumbar junction area from T10/11 to L1/2 can be collectively called thoracolumbar disc herniation (TLDH). The main symptoms of this disorder are neurological deficits, and high rates of disability have been widely reported [1–4]. Its surgical outcome is less satisfactory than is the case for herniations at lower lumbar levels [5]. The reasons for the suboptimal outcome are not fully understood, but in addition to the fact that the spinal canal is narrower at the thoracolumbar level and the spinal cord does not withstand much manipulation, two pathological characteristics of TLDH would definitely add risks to its surgical treatment. First, TLDH is frequently centrally located [1, 3, 4, 6]; second, they are known to frequently calcify and present as “hard discs” [1, 3, 4, 6]. Dickman reviewed 15 patients who had residual or incompletely excised symptomatic thoracic discs after their prior discectomies and found 13 of them had central calcified discs [7].

These central calcified compressive lesions are often large in volume [1] and propose high demand for wide surgical visualization during the operation. On the other hand, the premise of wide surgical exposure is that spinal stability must be well preserved. Therefore, traditional laminectomy was abandoned and anterior transthoracic approaches and several posterolateral/lateral approaches, which balance the benefit of surgical visualization and spinal stability, have been developed to treat TLDH [3, 4, 8]. The anterior transthoracic approach can be performed by minimally invasive thoracoscopic surgery [9–13]. However, it should be noted that the anterior approach and the posterolateral/lateral approaches, including costotransversectomy [8] and Larson’s extracavitory approach [4], are all essentially performed from one side and are best suitable for lateral, soft herniated discs. With central calcified discs, these approaches do not permit clear visualization and smooth excision of the lesion on the other side of the spinal canal that is not exposed, predisposing to incomplete decompression or inadvertent cord manipulation. Although in experienced hands, instruments can be sent to the other side of the spinal canal, “to reach there” is quite different from being able “to work there”. Moreover, these approaches involve seldom used incisions and manipulation of anatomical structures which are not familiar to spine surgeons [3, 4, 8]. And with the thoracoscopy approach, another problem is the difficulty in accessing the spinal levels below T11/12 caused by the diaphragm [11, 12].

We used a circumspinal decompression and fusion procedure through the conventional posterior midline incision to treat central calcified TLDH with neurological deficits. We chose the term “circumspinal” because the surgery involves laminectomy and bilateral resection of facet joints before removal of the herniated discs. This procedure offers a genuinely wide surgical view, and it allows bilateral, interactive manipulation of the central hard disc matter, thus facilitating decompression and reducing the risk of cord injury; moreover, the procedure is performed through an incision familiar to spine surgeons. However, this procedure requires wide resection of normal structures and, consequently, instrumentation and fusion to reconstruct stability. In this retrospective review, we report the clinical outcome we observed for this procedure with 2-year minimal clinical follow-up and radiological evaluation using reconstruction computed tomography (CT). Particular attention was paid to the improvement of neurological function, post-operative back pain and fusion.

## Materials and methods

### Patients

**Fig. 1:**
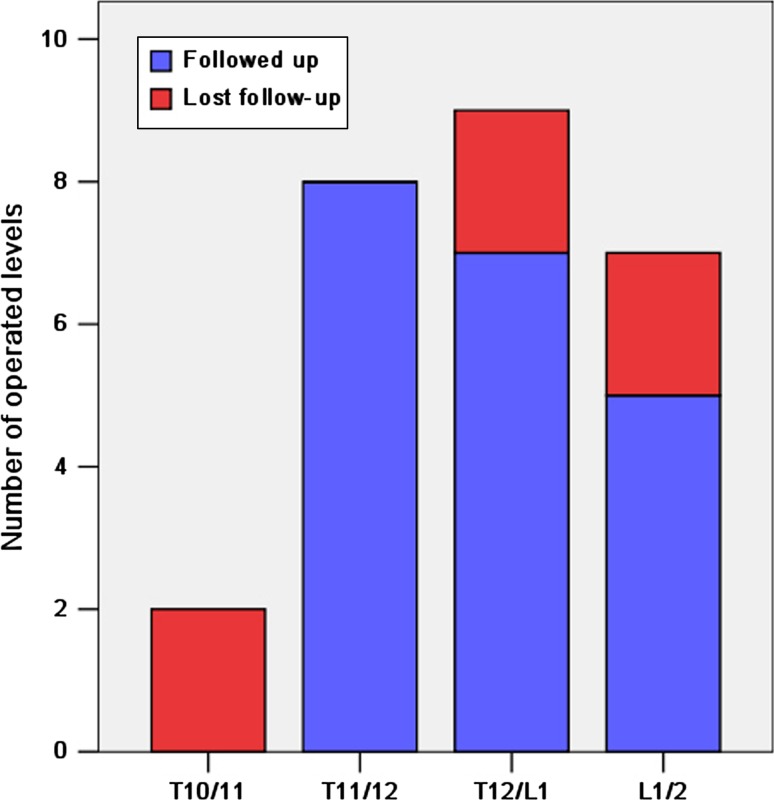
The distribution of the operated levels. A total of 26 discs were excised and six patients with single-level herniation (*red*) did not attend final follow-up

Between April 2008 and April 2011, 22 consecutive patients with central calcified TLDH underwent circumspinal decompression and fusion through a posterior midline incision in our institution. All of them were operated on for neurological deficits. These patients include 15 males and 7 females, with an average age of 49 years (range 25–77 years). The mean pre-operative duration of symptoms was 48.5 ± 42.8 months (range 1–144 months). Two patients had a previous history of laminectomy at lower lumbar levels. Six patients had a coexistent ossification or hypertrophy of the ligamentum flavum at the level involving the herniated disc. Physical examination revealed myelopathy in three patients, radiculopathy in nine patients and a combination of both in ten patients. Nine patients had sphincter dysfunction. (We did not have patients admitted because of pure back pain, primarily because in our country, most patients with axial discogenic pain would consider themselves to have “average suffering” rather than a “true disease” like paralysis, and consequently tend to accept conservative treatment rather than a major surgery.) Eighteen patients were operated on at a single level, and four patients were operated on at two levels. All the 26 herniated discs that were excised were central, that is, they were broad-based and extended across the midline significantly. In addition, all of them were “hard discs” that had a significant calcified component compressing the dura sac. The distribution of the operated levels is shown in Fig. 1. Before discharge, all 22 patients had been informed to visit our clinic at 3 months, 1 year and 2 years after the surgery. At the time of this study, all of them were invited to come back for follow-up again and 16 of them returned. These 16 patients had a mean follow-up period of 40.8 ± 9.3 months (range 24–57 months). Five of the remaining six patients were referred from a long distance from our institution, which perhaps explains why they did not return for this final follow-up. The information of the six patients who did not return for final follow-up is summarized in Fig. 1 and Table 1.

**Table 1:** Data of the six patients who did not attend final follow-up

	Sex	Age	Level	Time of last contact since the operation	Way of last contact	Neurological status at last contact	Back pain at last contact
1	Male	66	T10/11	51 months	Telephone interview	No change after surgery	Progressed from VAS 0 to 3
2	Male	64	T12/L1	30 months	Previous medical records	Initial improvement up to 18 months after surgery, but then developed lumbar neurogenic intermittent claudication that warranted another decompression from L1 to L5	Reduced from VAS 9 to 3
3	Male	77	T10/11	30 months	Previous follow-up charts	Mild improvement, mainly on working function and leg sensory	No back pain before surgery and at last contact
4	Female	46	L1/2	24 months	Previous follow-up charts	Significant improvement, mainly on walking function and leg sensory	No back pain before surgery and at last contact
5	Male	40	T12/L1	4 days	Previous medical records	Mild improvement, mainly on leg weakness	Mildly reduced
6	Female	35	L1/2	53 months	Telephone interview to her husband	Significant improvement, complete remission of leg pain, back to full-time work as a physician, she herself refused to respond because of “unhappy memories”. She was the patient who developed transient leg numbness and tarry stool after surgery	Complete remission

### Operative procedure

**Fig. 2:**
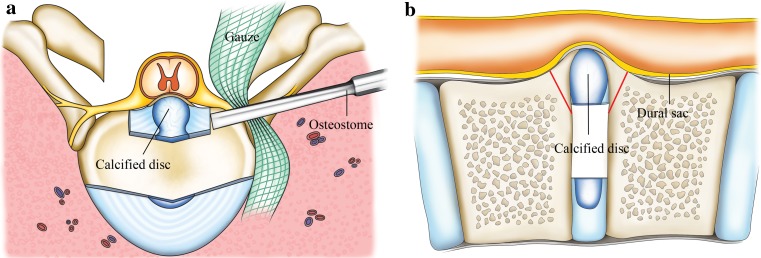
**a** After laminectomy and resection of the facet joint, the posterolateral rim of the herniated disc was exposed. After excision of the lateral portion of the disc, the osteostome was placed at the base of the hard disc to knock it off the connecting vertebral body before its final removal. **b** This is the sagittal view. The *red lines* indicate the position of the osteostome blade which was oriented to avoid the peak of the pathology that directly deforms the cord

**Fig. 3:**
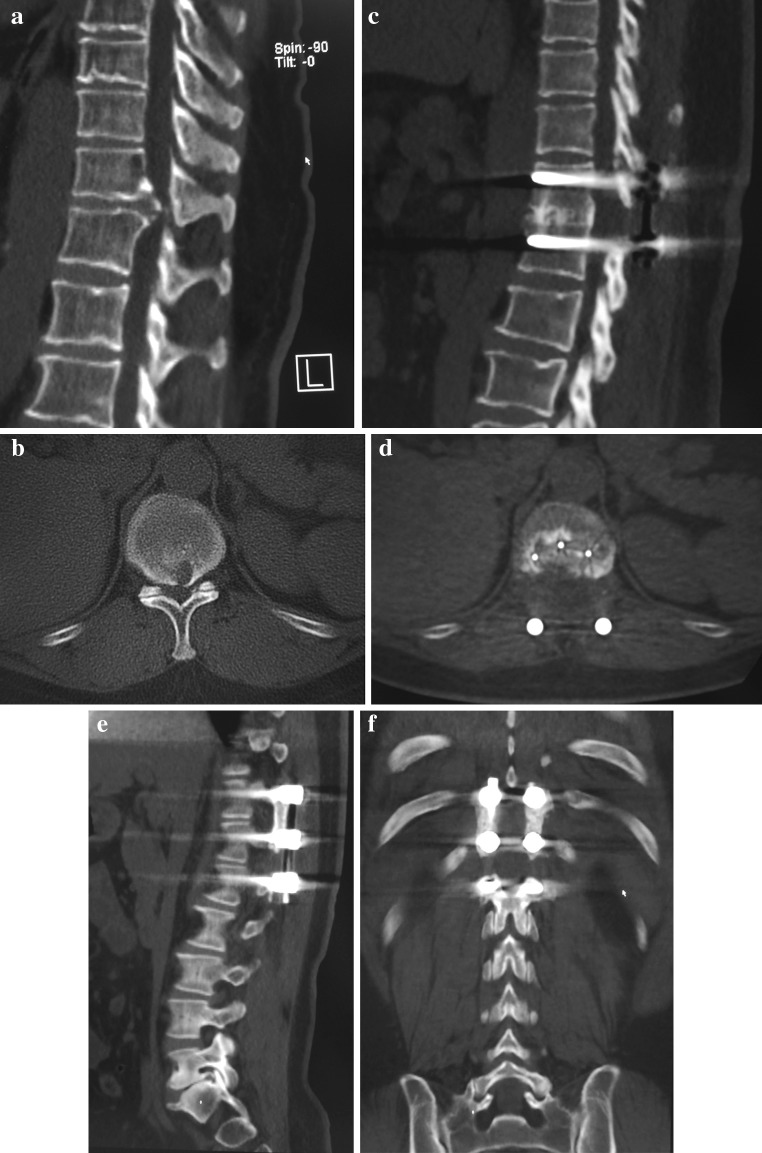
Pre-operative CT images (**a**, **b**) show the ventrally placed hard disc. Post-operative CT images (**c**, **d**) show that the herniated disc had been completely excised and the interbody fusion was solid. Images (**e**, **f**) demonstrate solid posterolateral fusion in another patient

In the prone position, the posterior elements were exposed through a midline incision. After instrumentation with bilateral pedicle screws at the segments of decompression, the laminectomy was performed. The ossification or hypertrophy of the ligamentum flavum, if present, was also removed. Next, the facet joints were resected in turn to make space for manipulating the disc. At this time, bleeding from the epidural venous plexus and vessels accompanying the exiting nerve root was usually massive, and it was necessary to coagulate the vessels carefully and identify the exiting nerve root and protect it with a small piece of cotton. Then, specially made smooth gauze was inserted into the gap between the pleura (or peritoneum) and the posterolateral surface of the disc to push the pleura away and make more space for discectomy (Fig. 2a). Curettes and disc rongeurs were used to remove the lateral portion of the disc first, leaving the middle portion of the disc for the next step. Commonly, the ventrally placed, calcified disc resembled a “hard shell” that bridged the adjacent vertebral bodies. We inserted a neural dissector into the gap between the base of the “hard shell” (the junction of the “shell” and the vertebral body) and the dura sac and gently separated the adhesion between them. Next, we placed the cutting edge of an osteostome on the base of the “hard shell”, avoiding the ridge/peak of it, which directly deformed the cord, and gently knocked the “hard shell” off of the vertebra from an angle as parallel to the horizon as possible (Fig. 2b). When this step of decompression was performed on one side, a rod was set into the screw heads on the other side for interim stability. After the “hard shell” was nearly isolated, a neural dissector was used again to dissect the compressive pathology off of the dura as the rongeur pulled it down into the interbody space before its final removal. Usually, successful ventral decompressions were achieved by alternative and interactive manipulation on the compressive lesion from both sides. Fusion surfaces were carefully prepared. In most cases, a cage with autogenous local bone was used for the fusion (Fig. 3). In cases with a narrow disc space, the disc space was closed by compressive instrumentation and posterolateral intertransverse/intercostal fusion was performed (Fig. 3). We did not use loupes, microscope or video-assisted endoscopy in the operation because we had not adapted to this equipment.

### Clinical outcome evaluation

The 16 patients who came back for the final follow-up were examined by an independent spine surgeon and then received plain radiographs and reconstruction CT examination at our institution. Although six patients did not return for the final follow-up, we managed to contact them by telephone or determine their situation through their medical records or previous follow-up charts. The clinical outcome evaluation items (Tables 2, 3). of this series included sixThe item “perioperative clinical data” covered all 22 patients and the other items only included the 16 patients who attended the final follow-up.

**Table 2:** Clinical outcome evaluation

Items of outcome evaluation	Outcome measures
Perioperative clinical data	Operative time, blood loss, perioperative complications and post-operative time of hospitalization
Neurologic status before surgery and at follow-up	A modified Japanese Orthopedic Association (JOA) scoring system (maximum 11 points) (Table 3)
Extent of decompression	Reconstruction CT, the results were rated either as “complete decompression” or “incomplete decompression”
Back pain before surgery and at follow-up	Linear visual analog scale (VAS), a scale with choices ranging from 0 (no pain) to 10 (intolerable pain)
Local spinal curvature before surgery and at follow-up	Local kyphotic angle which is equal to the included angle of the extension lines of the superior end-plate and the inferior end-plate of the fusion level
Fusion	Reconstruction CT. Fusion was confirmed if the following two criteria were both fulfilled: first, trabecular bone bridging was observed on the fusion surfaces on both the sagittal and coronal CT images; second, no instrumentation breakage was presented

**Table 3:** Modified Japanese Orthopedic Association (JOA) scoring system

Function score	Description
Motor	
Lower extremity	
0	Unable to stand up or walk by any means
0.5	Able to stand up but unable to walk
1	Unable to walk without a cane or other support on a level surface
1.5	Able to walk without a support but with a clumsy gait
2	Walks independently on a level surface but needs support on stairs
2.5	Walks independently when going upstairs, but needs support when going downstairs
3	Capable of fast but clumsy walking
4	Normal
Sensory	
Trunk	
0	Complete loss of touch and pain sensation
0.5	50 % of normal sensation or below and/or severe pain or numbness
1	Over 60 % of normal sensation and/or moderate pain or numbness
1.5	Subject numbness of a slight degree without any objective deficit
2	Normal
Lower extremity	
0	Complete loss of touch and pain sensation
0.5	50 % of normal sensation or below and/or severe pain or numbness
1	Over 60 % of normal sensation and/or moderate pain or numbness
1.5	Subject numbness of a slight degree without any objective deficit
2	Normal
Bladder function	
0	Urinary retention and/or incontinence
1	Sense of retention and/or dribbling and/or thin stream and/or incomplete continence
2	Urinary retardation and/or pollakiuria
3	Normal

Total score for a healthy person is 11

### Statistical analysis

The paired-sample *t* test was used to compare pre- and post-operative JOA scores and local kyphotic angles. The Wilcoxon signed-rank test was used to compare pre- and post-operative back pain VAS scores. SPSS 13.0 (SPSS Inc., IL, USA) software was used for data analysis and the α value was set at 0.05.

## Results

### Operative time, blood loss, post-operative time of hospitalization and perioperative complications

The mean operative time was 185.4 ± 42.4 min (range 92–253 min). The mean estimated blood loss was 895.5 ± 790.0 ml (range 300–4,000 ml). Blood loss exceeded 1,000 ml in 6 of the 22 patients and was less than 1,000 ml in the latest eight cases. The patient whose estimated blood loss was 4,000 ml was the eighth patient in this series and was operated on at two levels. In his operation, bleeding from the epidural venous plexus was severe and extremely difficult to coagulate. The mean post-operative time of hospitalization was 8.0 ± 2.2 days (range 6–15 days). The rate of perioperative complications was 18.2 % (4/22), and included transient deterioration of leg numbness and tarry stool in one patient, wound infection in one patient, leg deep venous thromboembolism in one patient and cerebrospinal fluid leakage in one patient, all of which resolved uneventfully.

### Neurological status

**Fig. 4:**
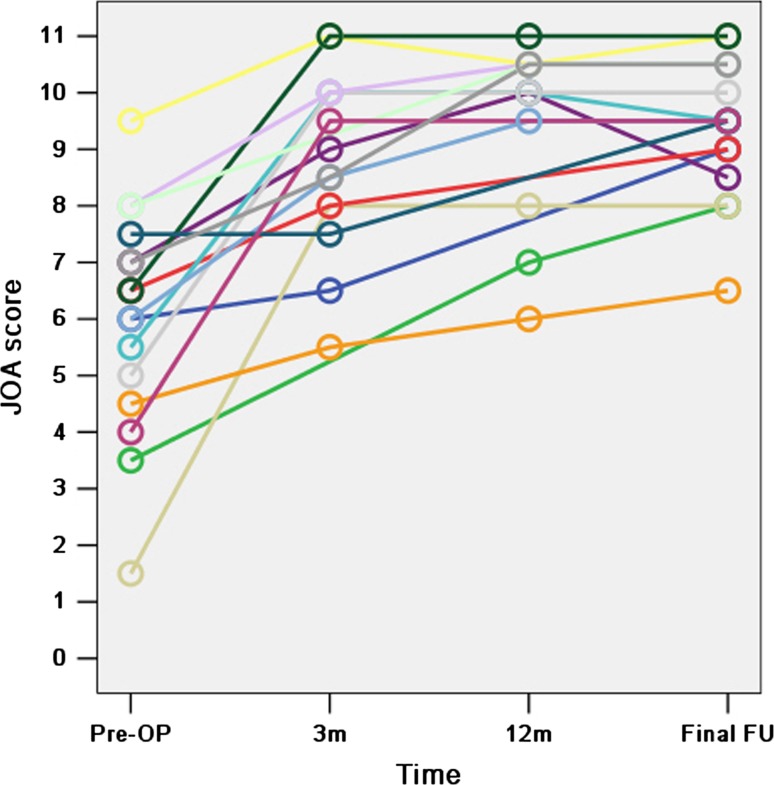
The *lines* represent the 16 patients who attended the final follow-up and the *small circles* represent the JOA score of the patients before surgery (Pre-OP), at 3 months (3 m), 12 months (12 m) after surgery and final follow-up (FU). Fourteen and 12 of the 16 patients were also reviewed 3 and 12 months after surgery at our clinic, respectively

All the 16 patients who attended the final follow-up improved after surgery. The mean JOA score before surgery and at the final follow-up was 6.0 ± 2.0 (range 1.5–9.5) and 9.4 ± 1.2 (range 6.5–11.0), respectively, and the difference between these values is statistically significant (*t* = −9.171, *P* = 0.000). It can be noted that major improvements of neurological status occurred within 3 months after operation, but improvements were seen up to 1 year (Fig. 4).

### The extent of decompression

The extent of decompression was rated as “complete decompression” in 12 patients and “incomplete decompression” in four patients, three of whom had a residual osteophyte on the vertebra and one of whom had a residual calcified fragment of the disc adhering to the dura. All these residual lesions were small and did not deform the neural elements.

### Back pain

The median back pain VAS score at final follow-up (median 1; range 0–9; lower quartile 0, upper quartile 2) was significantly lower than that before surgery (median 4; range 0–10; lower quartile 0, upper quartile 7.25) (*z* = −2.196, *P* = 0.028). Among the 16 patients, only one’s back pain progressed after surgery from a VAS of five before surgery to a VAS of nine at final follow-up. He was diagnosed at final follow-up with non-fusion by the reconstruction CT.

### Local spinal curvature

**Fig. 5:**
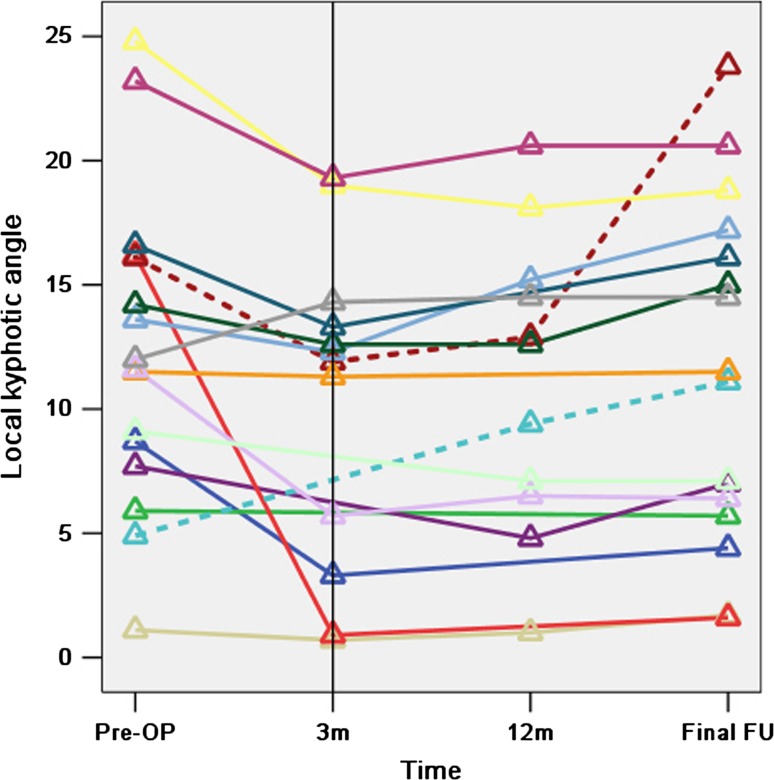
The *lines* represent the 16 patients who attended the final follow-up and the *small triangles* represent the local kyphotic angles of the fusion levels before surgery (Pre-OP), at 3 months (3 m), 12 months (12 m) after surgery and at final follow-up (FU). Twelve and 11 of the 16 patients who attended the final follow-up provided us with plain radiographs of the spine at 3 and 12 months, respectively. The two *dotted lines* represent the only two patients whose local kyphosis progressed significantly (more than 5°)

The mean local kyphotic angle at the fusion levels at final follow-up was 11.4° ± 6.9° (range 1.6°–23.8°). This was lower than that before surgery, which was 12.3° ± 6.3° (range 1.1–24.8°), but the difference is not statistically significant (*t* = 0.702, *P* = 0.493). Local kyphosis was reduced in nine patients and unchanged in one, and in six patients it progressed by 0.6°, 6.2°, 7.7°, 3.6°, 0.8° and 2.5°, respectively (Fig. 5). Two patients had a significant local kyphosis progression of more than 5°: one (6.2°, lower dotted line in Fig. 5) had a cage position close to the posterior rim of the vertebrae, and the other (7.7°, upper dotted line in Fig. 5) was a 74-year-old man whose local kyphosis progression was mainly due to degenerative narrowing of the operated disc space without interbody fusion in a follow-up period of 57 months. From Fig. 5, it can be noted that in most cases, the local kyphotic angle was notably reduced 3 months after surgery, but slight correction loss occurred in the long term.

### Fusion

In the 16 patients who attended the final follow-up, there were a total of 20 excised discs, and interbody fusion was performed at 14 levels, whereas posterolateral intertransverse/intercostal fusion was performed at six levels. According to our fusion assessment criteria based on reconstruction CT, 15 of the 16 patients had solid fusion at final follow-up, and the fusion rate was 93.8 %. In addition, no instrumentation breakage was noted. One patient was diagnosed as non-fusion because of discontinuous bone bridging and his axial CT image revealed a pedicle screw slightly perforating the inner pedicle wall. This patient had severe back pain (VAS score 9) when he was working. He was not reoperated upon but received conservative treatment.

## Discussion

This circumspinal decompression procedure is highly effective for decompression for central calcified TLDH, without excessive retraction of the cord or the exiting nerve roots. The goals of the operation were met by three technology points. First, the wide exposure provided by laminectomy and bilateral resection of the facet joints ensures that all the neural elements are under direct view of the surgeon throughout the discectomy process. Second, the disc was reached through a bilateral far-lateral approach and was resected by interactive, collaborative manipulation on both sides. Third, the central portion of the disc was first cut off at its base rather than at its peak, which directly compressed the cord. As a result, all of the 16 patients who attended final follow-up showed improvement at their final follow-ups. Complete decompression was achieved in 75 % of them, and the other four patients only had small residual lesions that did not deform the cord. As for the six patients who did not attend final follow-up, evidence of improvement at last contact was observed in five of them, and only one patient reported no change of symptoms. These data compare favorably with that in the literature. Kim et al. [2] reported the surgical results of the oblique paraspinal approach for disc herniation between T12/L1 and L2/3 and documented an overall patient satisfactory rate of 78.1 % at follow-up (mean follow-up, 28.1 months) in 19 patients. Gille et al. [1] reviewed 18 operated cases of hard thoracic disc herniation with 72 % of the lesions located between T8 and T12 and reported that 83 % of the patients had neurological improvement.

An 18.2 % (4/22) rate of perioperative complications was found in this series, which was slightly higher than the rate of 15.6 % found in Quint and Rosenthal’s series of 167 consecutive patients with thoracic disc herniation who underwent thoracoscopic surgery [11]. However, their cases include both soft and hard thoracic disc herniations [11]. In Gille’s series of 18 hard thoracic disc herniation patients, the rate of perioperative complications rose up to 61.1 % (11/18) and seven dural tears accounted for the majority of the perioperative complications [1]. Four of the seven cases of dural tears required later surgical revision. In contrast, in the current series of 22 central hard TLDH cases with neurological deficits, only two complications (temporary deterioration of leg numbness in one patient and cerebrospinal fluid leakage in another) were related to dural manipulation and both resolved with no adverse effect. This minimal violation to the dura was due to the wide exposure that allowed visualization of the dura throughout the decompression process.

The anterior transthoracic approach and lateral approaches, including Larson’s extracavitory approach and costotransversectomy, have been widely used for symptomatic TLDH [4, 8]. However, as mentioned earlier, these approaches are essentially one-sided approaches, and the neural elements on the other side of the spinal canal are not in direct view for most of the decompression process. With the anterior approach (transthoracic or retroperitoneal), it is necessary to access the dura through the compressive lesion, predisposing the patient to inadvertent cord injury. On the other hand, the anterior approach is technically demanding with various anatomic obstacles and it involves the use of thoracotomy, which requires entry into an unfamiliar territory where the control of massive bleeding and cerebrospinal fluid leakage would be very difficult [1]. In addition, the anterior approach is associated with several respiratory complications and approach-related morbidities [3, 9–12]. Thoracoscopy has been developed as a minimally invasive variant to thoracotomy. As Rosenthal et al. [11, 12] reported, thoracoscopy provided identical visualization of the pathology, with significantly fewer approach-related morbidities, less pain, fewer pulmonary problems and shorter hospitalizations. These authors also demonstrated that complete and safe decompression can be achieved by thoracoscopy in some cases with large hard thoracic disc herniation [11]. However, it should be noted that performing thoracoscopy is difficult for pathology below the level of T11/12; furthermore, this procedure is a highly specialized technique that only a limited number of spine surgeons with endoscopy backgrounds are able to offer to their patients. With Larson’s extracavitary approach and costotransversectomy, both of those procedures require removal of one or two sufficiently long pieces of rib and extensive displacement of the pleura to secure a lateral visualization to resect the ventrally placed central lesion [4, 8]. Therefore, they are still associated with the risk of pulmonary complications [3, 12]. As an alternative, the circumspinal decompression procedure does not violate the respiratory system. The herniated disc is accessed from a far-lateral approach on both sides and excised by a collaborative manipulation from both sides. Therefore, the circumspinal decompression procedure does not require generous removal of ribs or extensive dissection of the pleura. In this series, none of the observed complications involved the respiratory system, and no patient needed a chest tube. The advantages of this procedure also include the use of a familiar surgical incision, familiar surgical techniques and familiar surgical instruments.

It is thoroughly important to note that the circumspinal decompression and fusion procedure is a major and destructive procedure. From our results, the majority of the complications in the current series including tarry stool (most likely stress ulcer), wound infection and deep venous thromboembolism are all associated with the effects of a major surgery. The basic philosophy of this procedure was that to generate wider visualization, we must, unfortunately, remove more normal structures. However, we did not extend the instrumentation to healthy levels, and CT-assessed solid fusion was achieved in all but one patient who had a malpositioned pedicle screw that could explain the non-fusion. On the other hand, overall back pain did not progress, but, rather was significantly alleviated at final follow-up (including the six patients who did not attend final follow-up, Table 1) and local spinal curvature was not significantly altered. Although a few mild losses of local kyphosis correction were observed during the follow-up period, this is in agreement with the general law of thoracic interbody fusion [14]. In general, spinal stability was well maintained in the long term by instrumentation and fusion. In addition, although this procedure carries a risk of injuring the artery of Adamkiewicz that usually arises between T9 and L2 and supplies the anterior spinal cord, no post-operative ischemic myelopathy was observed in this series, possibly because these patients only had one or two operated levels and the operation did not include a corpectomy.

Various surgical approaches have been developed to treat symptomatic TLDH. The authors of this article believe that it may not be wise to elect a “best” approach because patients’ conditions are diversified, and each approach has its unique advantages and disadvantages. The circumspinal decompression and fusion procedure provides an extremely wide visualization but requires extensive resection of anatomical structures. The indication of this procedure is and should be limited to central calcified TLDH patients with neurological deficits. For patients with refractory pure back pain, we had no surgical experience, but we would prefer discectomy and fusion through an anterior transthoracic approach or thoracoscopic surgery. This procedure would be a particularly suitable option for patients with pulmonary morbidity that contradicts a thoracotomy or for spine surgeons who are most familiar with the conventional posterior approach. Although we present a monocenter clinical report with a limited number of cases, we believe that spine surgeons should be made aware of this procedure.
